# Factors associated with potentially missed acute deterioration in primary care: cohort study of UK general practices

**DOI:** 10.3399/BJGP.2020.0986

**Published:** 2021-06-02

**Authors:** Elizabeth Cecil, Alex Bottle, Azeem Majeed, Paul Aylin

**Affiliations:** Department of Primary Care and Public Health, Imperial College London.; The Dr Foster Unit, Department of Primary Care and Public Health, Imperial College London, London.; Department of Primary Care and Public Health, Imperial College London.; The Dr Foster Unit, Department of Primary Care and Public Health, Imperial College London, London.

**Keywords:** emergency hospital admissions, health deterioration, patient safety, primary care, sepsis, urinary tract infections

## Abstract

**Background:**

In the UK, while most primary care contacts are uncomplicated, safety incidents do occur and result in patient harm, for example, failure to recognise a patient’s deterioration in health.

**Aim:**

To determine the patient and healthcare factors associated with potentially missed acute deterioration in health.

**Design and setting:**

Cohort of patients registered with English Clinical Practice Research Datalink general practices between 1 April 2014 and 31 December 2017 with linked hospital data.

**Method:**

A potentially missed acute deterioration was defined as a patient having a self-referred admission to hospital having been seen in primary care by a GP in the 3 days beforehand. All diagnoses and subsets of commonly-reported missed conditions were analysed..

**Results:**

A total of 116 097 patients contacted a GP 3 days before an emergency admission. Patients with sepsis (adjusted odds ratio [aOR] 1.09, 95% confidence interval [CI] = 1.01 to 1.18) or urinary tract infections (aOR 1.09, 95% CI = 1.04 to 1.14) were more likely to self-refer. The duration of GP appointments was associated with self-referral. On average, a 5-minute increase in appointment time resulted in a 10% decrease in the odds of self-referred admissions (aOR 0.90, 95% CI = 0.89 to 0.91). Patients having a telephone consultation (compared with face-to-face consultation) (aOR 1.14, 95% CI = 1.11 to 1.18) previous health service use, and presence of comorbidities were also associated with self-referred admission.

**Conclusion:**

Differentiating acute deterioration from self-limiting conditions can be difficult for clinicians, particularly in patients with sepsis, urinary tract infections, or long-term conditions. The findings of this study support the call for longer GP consultations and caution against reliance on telephone consultations in primary care; however, more research is needed to understand the underlying mechanisms.

## INTRODUCTION

In the UK, GPs working for the NHS provide most first-contact health care,^1^ with >300 million primary care consultations conducted annually in England alone.^2^ Most contacts are harm-free; however, around 2% of patients will experience a safety incident during their care.^3^ While some incidents, such as unexpected complications during the provision of correct care, are not preventable, others, such as failure to recognise serious illness or patient deterioration, can contribute to avoidable harm.^4^

Investigations into the determinants of missed acute deterioration in primary care are limited. A single study from the UK,^5^ which investigated delayed escalation of care in deteriorating patients, focused on out-of-hours primary care provision and did not link to data on other healthcare contacts. Studies investigating diagnostic errors are more common, where most are considered to take place during patient assessment,^6^^–^^8^ and the presence of comorbidity often contributes.

This study aimed to investigate the factors related to self-referral to hospital in acutely deteriorating patients who had previously visited a GP, for all conditions and for four commonly missed diagnoses in primary care:^9^ pulmonary embolism, urinary tract infections, ectopic pregnancies, and sepsis. Primary and secondary care linked data were used as a novel approach to recognise potentially missed deterioration in primary care.

## METHOD

### Study design

A population-based observational study was conducted to investigate patient journeys through primary care to treatment in hospital. Acute deterioration, the worsening of a patient’s condition towards critical illness, was measured as an emergency hospital admission.

### Data sources

The Clinical Practice Research Datalink (CPRD) is a validated, nationally representative primary care database of patient-level, longitudinal health records, covering 7% of the UK population.^10^ The CPRD is linked to Hospital Episode Statistics, containing inpatient and emergency department activity, in NHS hospitals in England. (The datasets analysed during the current study are not publicly available as access is subject to approval. The authors will consider requests for data sharing on an individual basis; however, they will be governed in respect of data sharing by the data owners (the CPRD) and any requests to share will be subject to their permission, and to the approval of ethics committees overseeing the use of these data sources.)

### Population

The study cohort consisted of patients of all ages, who experienced an acute deterioration in health between 1 April 2014 and 31 December 2017 in England. An acute deterioration in health was defined as an emergency hospital admission (as opposed to an elective or planned admission). Patients were selected who had been registered with a CPRD practice for at least a year. Any admissions that were readmissions within 3 days of the index admission were excluded. Subgroups were created for patients admitted for four specific conditions that are reportedly commonly missed in primary care.^9^ International Classification of Diseases 10th Revision (ICD-10) codes identified emergency admissions for pulmonary embolism, urinary tract infection or pyelonephritis, ectopic pregnancies, and sepsis (see Supplementary Table S1 for all codes used in this study).

**Table:** How this fits in

Failure to recognise serious illness (or patient acute deterioration) can contribute to avoidable harm to a patient. Little is known about the determinants of missed acute deterioration in primary care. This study found shorter GP consultations or those via telephone were associated with potentially missed acute deterioration. These findings are highly relevant to clinicians as GP telephone and video consultations have increased substantially with the COVID-19 pandemic. These forms of consultation need to be fully evaluated to support a safe primary care.

### Primary care consultations

The CPRD provides information on consultation type, staff members, and clinical information from general practice. Consultations in the CPRD represent occasions when a patient’s electronic health record is opened. The duration recorded is the length of time the health record is open.^11^

Primary care consultations were investigated in the 3 days (0–2 days) before hospital admission because a patient’s acute deterioration is likely to be apparent within this time (personal communication, A Majeed 2020). An assumption was made that it would be possible to determine health deterioration, even in a single consultation. If a patient had >1 primary care visit, data were investigated for the last consultation before hospital admission. Consultations were classified as face-to-face or via telephone, and with a GP or nurse. A patient’s number of primary care consultations was calculated (excluding those within 3 days of the index admission) during the 12 months leading up to the admission (see Supplementary Tables S2 and S3 for coding).

### Hospital use in the 12 months before admission

The number of emergency department visits within the past 12 months was categorised into 0, 1, or 2+. Previous admissions within 30 days of the index admission were categorised into surgical and non-surgical (see Supplementary Table S4 for surgical codes). Emergency admissions and planned/elective admissions in the 12 months (excluding those within 30 days of the index admission) before admission were categorised into 0 or 1+.

### Patient demographic factors

Covariates were identified from previous studies known to increase the risk of an emergency admission: age,^12^ sex, morbidity, and level of deprivation.^13^ The presence of long-term conditions was determined from coding in the patient’s primary care records before the admission (see Supplementary Table S5 for coding). Patients’ socioeconomic status, based on residential postcode, was derived from linked Indices of Multiple Deprivation data 2015.^14^

### Outcome

The study focused on patients who consulted a GP and excluded consultations with other healthcare professionals. A patient who is deteriorating who consults a GP either has the acute deterioration recognised and is referred (to the emergency department, directly to hospital, or to another healthcare service); or the patient subsequently visits the emergency department as a self-referral.

**Figure 1.**
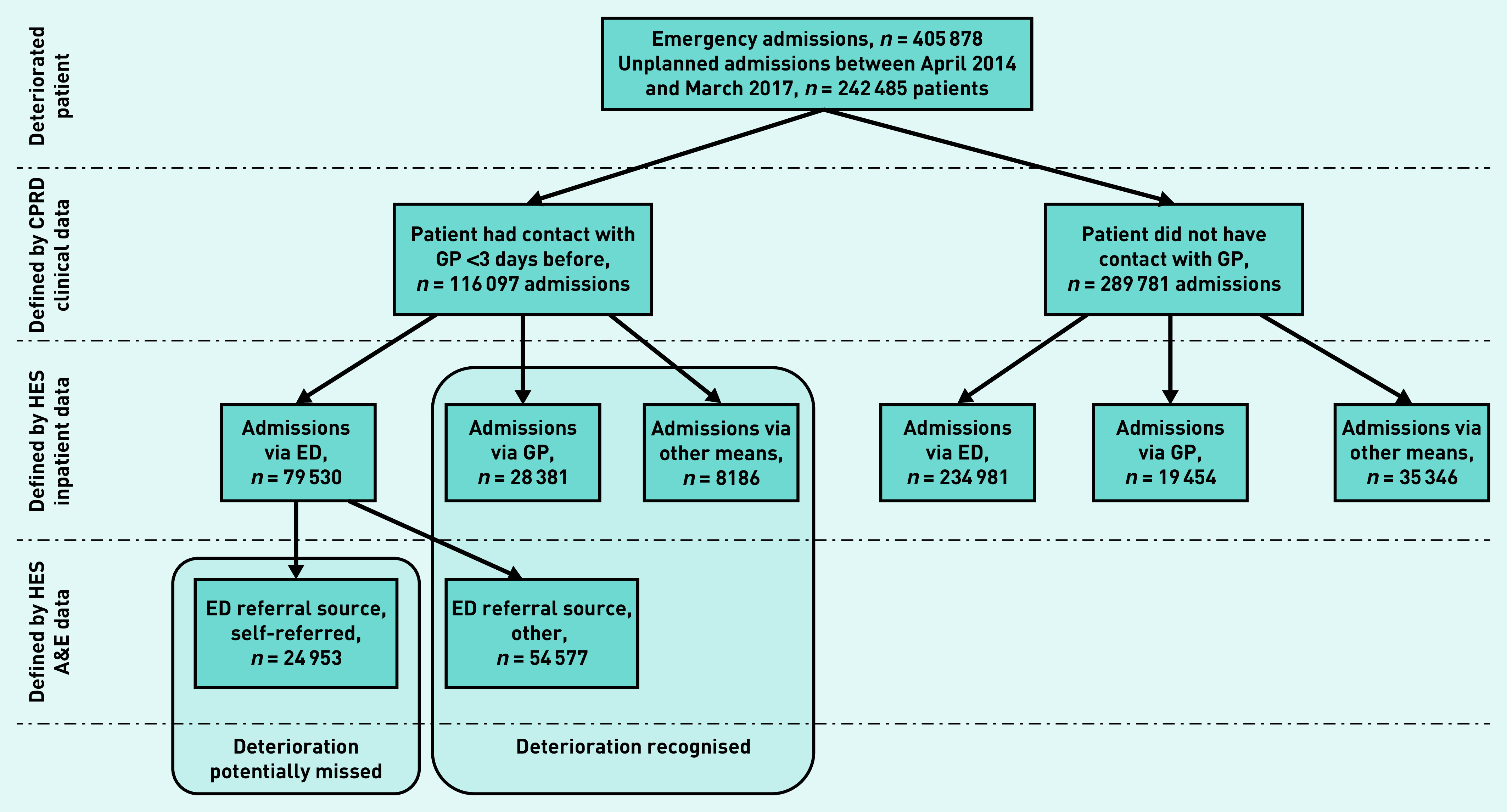
*Definition of potentially missed acute deterioration by GPs using primary and secondary care linked data. A&E = accident and emergency. CPRD = Clinical Practice Research Datalink. ED = emergency department. HES = Hospital Episode Statistics.*

A potentially missed acute deterioration was defined as a patient who had been seen in primary care by a GP in the 3 days before hospitalisation, having a self-referred admission (an emergency admission via the emergency department and corresponding self-referred emergency department visit) (Figure 1 and see Supplementary Tables S6 and S7 for coding). The primary outcome was self-referred admission to hospital.

### Statistical analysis

Logistic regression was applied with generalised estimating equations, clustered by GP practice, assuming an exchangeable correlation structure. Multivariable analyses (backwards selection) and a sensitivity analysis were carried out excluding consultations <5 min (those more likely to have misclassified consultation types). Stata (version 15) was used for the analyses.

## RESULTS

Of the 3 089 403 patients registered at all CPRD participating practices in England (280 practices), there were 405 878 emergency admissions by 242 485 patients over the study period (Figure 1). Of these admissions, 564 were for ectopic pregnancy, 2407 were for pulmonary embolism, 5383 were for sepsis, and 15 015 were for urinary tract infections (see Supplementary Table S8 for details). Most admissions were through the emergency department (77.5%, *n* = 314 511/405 878), and 11.8% (*n* = 47 835/405 878) were via a GP. In total, 10.7% (*n* = 43 532/405 878) of admissions were via other means (for example, an emergency admission via consultant clinic or via another healthcare provider) (Figure 1). On linking datasets, 96.4% (*n* = 303 074/314 511) of emergency department admissions had a corresponding emergency department visit record (data not shown).

### Contact with primary care in the 3 days before an emergency admission

Around one in three (*n* = 127 197/405 878) patients had contact with primary care in the 3 days before admission; most consultations (91.3%, *n* = 116 097) were with a GP (data not shown). The proportion of patients who self-referred varied across regions: for example, 11.8% of patients (*n* = 1721/14 641) self-referred in the South West region compared with 30.7% of patients (*n* = 4189/13 639) in London (Table 1). Of those who consulted with a GP in the 3 days before admission, 20.0% (*n* = 23 232/116 097) had >1 contact in primary care (see Supplementary Table S9 for details). Most patients (87.0%, *n* = 101 014/116 097) had face-to-face contact; 19.8% (*n* = 23 039) were telephone contacts; and 4.7% (*n* = 5442) were out-of-hours contacts. Of patients who had face-to-face contact with a GP, 10.4% (*n* = 10 483/101 014) also had a coded ‘telephone encounter’ (Supplementary Table S10). Primary care consultations lasted for a median of 9 min (interquartile range [IQR] 3–18).

**Table 1.** Patient and condition characteristics of emergency admissions to hospital between April 2014 and December 2017 by patients’ GP engagement and admission referral^a^

**Variable**	**Self-referred,^b^ *n* (%)**	**Other, *n* (%)**	**Total, *N***
All unplanned admissions	24 953 (21.5)	91 144 (78.5)	116 097

Ectopic pregnancy	14 (13.2)	92 (86.8)	106

Pulmonary embolism	161 (19.0)	688 (81.0)	849

Sepsis	410 (26.5)	1136 (73.5)	1546

UTI	1273 (25.2)	3784 (74.8)	5057

**Sex**			
Male	11 105 (21.4)	40 699 (78.6)	51 804
Female	13 847 (21.5)	50 443 (78.5)	64 290

**Deprivation level^c^**			
1 (least deprived)	4988 (19.2)	21 005 (80.8)	25 993
2	4923 (21.2)	18 341 (78.8)	23 264
3	5242 (21.3)	19 316 (78.7)	24 558
4	5213 (23.8)	16 667 (76.2)	21 880
5 (most deprived)	4575 (22.5)	15 783 (77.5)	20 358

**GP recorded comorbidity**			
No comorbidity	4431 (20.0)	17 746 (80.0)	22 177
1	5404 (21.8)	19 395 (78.2)	24 799
2+	15 118 (21.9)	54 003 (78.1)	69 121

**Region**			
East Midlands	3 (7.5)	37 (92.5)	40
East of England	2427 (24.9)	7314 (75.1)	9741
London	4189 (30.7)	9450 (69.3)	13 639
North East	309 (11.3)	2417 (88.7)	2726
North West	3991 (22.5)	13 734 (77.5)	17 725
South Central	2891 (16.8)	14 362 (83.2)	17 253
South East Coast	6484 (29.3)	15 639 (70.7)	22 123
South West	1721 (11.8)	12 920 (88.2)	14 641
West Midlands	2599 (16.5)	13 195 (83.5)	15 794
Yorkshire	339 (14.0)	2076 (86.0)	2415

	**Median (IQR)**	**Median (IQR)**	**Median (IQR)**

Patient age, years	67 (42–81)	66 (41–81)	66 (41–81)

a *The table displays row percentages. Data are for 116 097 emergency admissions by 90 193 patients who had a consultation with a GP between April 2014 and December 2017.*

b *Self-referred admission is defined as an admission via the emergency department and a corresponding emergency department record with a referral source of self-referred.*

c *Indices of Multiple Deprivation population weighted fifths. IQR = interquartile range. UTI = urinary tract infections.*

### Health service use in the 12 months before emergency admission

In 19.5% of admissions (*n* = 22 596/116 097), the patient had had a previous hospital (surgical or non-surgical) admission within 30 days. Patients who self-referred had, on average, higher rates of primary care consultations, emergency department visits, and emergency admissions in the previous 12 months than those referred by a GP (Table 2).

**Table 2.** Emergency department contact and previous health contacts in patients who consulted a GP in the 3 days before an emergency admission to hospital for all diagnoses and commonly missed conditions, by admission referral^a^

**Variable**	**Self-referred,^b^ *n* = 24 953, *n* (%)**	**Other referred, *n* = 91 144, *n* (%)**	**Total, *N* = 116 097, *n***
**ED visits (3–365 days)**			
0	9628 (18.2)	43 370 (81.8)	52 998
1	6032 (21.6)	21 859 (77.4)	27 891
2+	9293 (26.4)	25 915 (73.6)	35 208

**A previous hospital admission <30 days**			
For a surgical procedure	2274 (24.3)	7079 (75.7)	9353
Non-surgical admission	3279 (24.8)	9964 (75.2)	13 243

**Previous hospital admission (30–365 days)**			
Elective 1+	6696 (22.5)	23 111 (77.5)	29 807
Emergency 1+	9992 (23.5)	32 516 (76.5)	42 508

	**Mean (95% CI)**	**Median (IQR)**	**Mean (95% CI)**

**Primary care consultations (3–365 days)**			
GP	9 (4 to 17)	8 (4–5)	8 (4 to 16)
Nurse	2 (0 to 4)	2 (0–4)	2 (0 to 4)
Face-to-face	11 (5 to 19)	10 (5–18)	10 (5 to 18)
Telephone	0 (0 to 2)	0 (0–2)	0 (0 to 2)

a *Data are for 116 097 emergency admissions by 90 193 patients who had a consultation with a GP between April 2014 and December 2017.*

b *Self-referred admission is defined as an admission via the emergency department and a corresponding emergency department record with a referral source of self-referred. ED = emergency department. IQR = interquartile range.*

### Determinants of GPs who potentially missed acute deterioration in patients

Women admitted to hospital as an emergency with ectopic pregnancy had lower odds of self-referred admission compared with other conditions (adjusted odds ratio [aOR] 0.59, 95% confidence interval [CI] = 0.37 to 0.94) (Table 3). Patients admitted with sepsis (aOR 1.09, 95% CI = 1.01 to 1.18) or urinary tract infections (aOR 1.09, 95% CI = 1.04 to 1.14) were more likely to self-refer. Older patients were slightly less likely to self-refer. Older patients were slightly less likely to self-refer (aOR 0.99, 95% CI = 0.99 to 1.00) with each 10-year age increase. Patients with a GP-reported comorbidity were more likely to self-refer (aOR 1.07, 95% CI = 1.03 to 1.11) (Table 3). GP consultation factors associated with self-referral were the number of consultations in the 3 days before admission, type of consultation, consultation duration, and sex of GP.

**Table 3.** Crude and adjusted odds ratios comparing odds of a self-referred admission in patients who visited a GP in the 3 days before emergency admission^a^

**Variable**	**Unadjusted, *N*= 116 097, OR (95% CI)**	**Adjusted, *N*= 116 097, OR (95% CI)**
**Admission diagnosis**		
Ectopic	0.57 (0.36 to 0.90)	0.59 (0.37 to 0.94)
Sepsis	1.15 (1.06 to 1.24)	1.09 (1.01 to 1.18)
Pulmonary embolism	0.88 (0.78 to 1.00)	—
UTI	1.14 (1.09 to 1.19)	1.09 (1.04 to 1.14)
All other	*Reference*	*Reference*

**Patient factors**		
Sex, women versus men	1.00 (0.98 to 1.02)	—
Age, 10-year increase	1.01 (1.01 to 1.02)	0.99 (0.99 to 1.00)

**Indices of Multiple Deprivation**		
Most versus least deprived	1.02 (0.98 to 1.06)	—

**GP reported comorbidity**		
None	*Reference*	*Reference*
1	1.09 (1.05 to 1.12)	1.07 (1.03 to 1.11)
2+	1.10 (1.07 to 1.12)	1.05 (1.01 to 1.08)

**Health service use in patients who are deteriorating within 3 days**		
Number of GP consultations	0.91 (0.88 to 0.93)	0.91 (0.89 to 0.93)

**Consultation type**		
Face-to-face	*Reference*	*Reference*
Telephone	1.26 (1.22 to 1.30)	1.14 (1.11 to 1.18)
Face-to-face and telephone	0.90 (0.86 to 0.94)	0.89 (0.85 to 0.92)

**GP consultation duration**		
5-minute increase	0.89 (0.89 to 0.90)	0.90 (0.89 to 0.91)
Female GP	0.93 (0.91 to 0.98)	0.96 (0.94 to 0.98)
Also seen by a nurse	0.84 (0.80 to 0.87)	0.89 (0.85 to 0.92)

**Health service use in previous 12 months**		
Number of GP consultations, 3–365 days	1.01 (1.01 to 1.01)	—

**Admission in previous 3-<30 days**		
None	*Reference*	*Reference*
Surgical	1.16 (1.12 to 1.20)	1.07 (1.04 to 1.11)
Non-surgical	1.16 (1.13 to 1.20)	1.03 (1.00 to 1.06)

**ED visits previous (3–365 days)**		
0	*Reference*	*Reference*
1	1.14 (1.11 to 1.17)	1.13 (1.10 to 1.16)
2	1.31 (1.28 to 1.34)	1.29 (1.25 to 1.33)

**Previous admissions (30–<365 days)**		
Elective (1+ versus 0)	1.05 (1.02 to 1.07)	—
Emergency (1+ versus 0)	1.13 (1.12 to 1.16)	0.94 (0.91 to 0.96)

a *Logistic model using generalised estimating equations, clustering by GP practice, for 116 097 emergency admissions by 90 193 patients. ED = emergency department. OR = odds ratio. UTI = urinary tract infections.*

Patients who consulted with a female GP were 4% less likely to self-refer (aOR 0.96, 95% CI = 0.94 to 0.98), while patients who had a face-to-face followed by a telephone consultation with a GP in the 3 days before an emergency admission were less likely to self-refer (aOR 0.89, 95% CI = 0.85 to 0.92). With a 5-min increase in GP consultation length, there was a 10% decrease in the adjusted odds of self-referred admission (aOR 0.90, 95% CI = 0.89 to 0.91). Previous health service use was associated with self-referral: previous non-surgical admission (aOR 1.03, 95% CI = 1.00 to 1.06) or surgical procedure (aOR 1.07, 95% CI = 1.04 to 1.11) within 30 days; or having visited the emergency department (aOR 1.13, 95% CI = 1.10 to 1.16) in the past 12 months (Table 3). Previous GP consultation rate was not associated with a self-referred admission.

### Sensitivity analysis

In total 33.6% (*n* = 39 010/116 097) of GP consultations lasted <5 min. Adjusted odds ratios were similar after excluding these short consultations (see Supplementary Table S11 for details).

### Specific conditions

One in five women (*n* = 106/564) who were admitted as an emergency for ectopic pregnancy had had contact with a GP in the 3 days before admission. Similarly, 35.3% (*n* = 849/2407) of patients with pulmonary embolism, 28.7% (*n* = 1546/5383) of patients with sepsis, and 33.7% (*n* = 5057/15 015) of patients with urinary tract infections had had contact with a GP in the 3 days before admission (Table 1). In total, 13.2% (*n* = 14/106) of women with ectopic pregnancy self-referred, as did 19.0% (*n* = 161/849) of patients with pulmonary embolism, 26.5% (*n* = 410/1546) of patients with sepsis, and 25.2% (*n* = 1273/5057) of patients with urinary tract infections (Table 1). Consultation duration was consistently shorter in patients who self-referred across all conditions. A 5-min increase in consultation time was associated with an 11% (aOR 0.89, 95% CI = 0.81 to 0.98) decrease in the odds of self-referral in patients with pulmonary embolism, a 9% (aOR 0.91, 95% CI = 0.85 to 0.96) decrease in patients with sepsis, and a 7% (aOR 0.93, 95% CI = 0.91 to 0.96) decrease in patients with urinary tract infections. For patients with pulmonary embolism there was evidence that procedure within 30 days was positively associated with self-referral (aOR 1.59, 95% CI = 1.07 to 2.34) (see Supplementary Table S12).

## DISCUSSION

### Summary

One in three patients in this study were found to have had contact in primary care in the 3 days before hospital admission. In patients who had seen a GP, the proportion of patients with potentially missed acute deterioration (those who self-referred) varied across regions, age groups, conditions, and patients’ comorbidities. Patients who self-referred had had a significantly shorter consultation duration in primary care. Patients with sepsis or urinary tract infections (compared with other conditions) were 9% more likely to self-refer. The duration of GP appointments was negatively associated with a self-referral, which might suggest that longer appointments in which to assess patients could help improve patient safety and clinical outcomes. Previous health service use and telephone consultations were also associated with a self-referred admission.

### Strengths and limitations

To the authors’ knowledge, this is the first study to investigate the factors relating to GP referral in patients who are acutely deteriorating using linked primary and secondary care data. Recognition of clinical deterioration and immediate management of the condition includes referral to secondary care.

The size of the cohort, at almost 250 000 patients, means that the findings are unlikely to be due to chance. However, there may be inherent biases. For example, selection bias due to inclusion/exclusion criteria means that this study may not represent more transient populations. Small numbers were found for rare conditions: for example, of 106 women with ectopic pregnancy who had had contact with a GP, only 14 had a self-referred admission.

This study is reliant on coding by GP practice and hospital staff, and subject to biases of misclassification and missing data. GPs must remember to change consultation type when administrative tasks are performed, for example, when entering test results of a patient who is not present. A study using video recording of 229 GP consultations found that consultation duration ranged between 2 and 30 min.^16^ For the current study, it was assumed that administrative tasks would take less than 5 min per patient. The omission of GP consultations of <5 min (sensitivity analysis) had little impact on estimated associations.

This study assumes that a patient who visits a GP yet self-refers to hospital as an emergency has, potentially, had their acute deterioration missed. There are challenges in defining and measuring missed diagnoses in primary care. Disease is often self-limiting, yet in certain serious conditions, such as meningitis, disease progression can be rapid. Primary care clinicians need to aim for a balance between over-diagnosis and under-diagnosis.^17^ Previous research has found that diagnostic errors are often preceded by common symptoms and common, relatively benign, initial diagnoses.^18^ Consultations are likely to be accompanied by safety-netting advice, and the subsequent ‘telephone encounters’ found in this study recorded in the health records of patients who are deteriorating may suggest that patients are being monitored. Notably, patients who had both face-to-face and telephone consultations were found to be less likely to self-refer than those who had only one type of consultation. More research is needed to investigate these contacts further. This study found that the proportion of patients who self-referred to hospital following contact with a GP was highly variable between regions. This variation may be explained by GP practice factors such as the ability to contact a GP,^19^^,^^20^ or by a hospital’s policy on recording the method of admission. The study’s use of a multilevel model clustering by GP practice means that it investigated within practice variation; therefore, GP practice factors such as access will be controlled for. However, the behaviours of individual GPs are not.

### Comparison with existing literature

The emergency admission rates (see Supplementary Box S1 for details) for all valid patients registered with the CPRD partnered practices are comparable with those found in previous studies.^21^^–^^23^ GP consultation rates in the current study’s cohort are slightly higher than rates previously reported;^24^ however, it is not surprising because the cohort only includes patients with an emergency admission over the study period, and such patients may be generally sicker than the general population.

Although the effect size was small, the study found, after adjusting for confounders, that older patients were less likely to self-refer (1% less likely with each 10-year increase in age). Previous work that investigated the factors associated with a risk of delayed escalation in out-of-hours primary care^5^ found a positive association between age and delayed escalation. The current study did not control for GP diagnosed morbid conditions, which may explain the difference between these findings.

Surgery is known to be a strong predictor of emergency hospital admission, particularly for certain conditions such as pulmonary embolism. The present study confirms this. After surgery, patients will be discharged back to primary care with followup consultations in outpatient departments. A clear plan from the discharging surgical team must be conveyed to GPs and patients if they are to be truly efficient at spotting acute deterioration.

A previous investigation into patient safety incident reports in England and Wales found that failure to recognise signs of clinical deterioration, resulting in delayed management, was a major factor in serious harm-related incidences in primary care.^15^ Differentiating acute deterioration from self-limiting conditions can be difficult for clinicians, particularly for patients with sepsis or urinary tract infections, or with GP recorded long-term conditions.

### Implications for research and practice

An average GP consultation in the UK lasts 10 min,^25^ yet there has been a call for 15-min consultations to allow for *‘improved decision making and case management’*.^26^ This study found that patients who had longer consultations with their GP were less likely to have a subsequent self-referred admission. This might be because GPs with more time to assess patients are more likely to recognise deterioration and refer the patient for secondary care. It may also allow GPs more time to provide advice such as to contact the GP again if their condition worsens. This study found a patient who has a face-to-face appointment followed by a telephone contact is 11% less likely to self-refer; however, the findings need to be interpreted with caution. This may be an example of reverse causation. A GP who recognises acute deterioration in a patient will either refer the patient directly to hospital or call the emergency department to warn them that the patient is on their way. The act of contacting a hospital will add time to the consultation and could explain the described association. Further research is required to understand the mechanisms.

Increasing consultation times may also decrease GPs’ workload overall.^11^ Certainly, increasing consultation times would allow GPs more time to engage with the national early warning scores (NEWS),^27^ a structured way of communicating the severity of a patient’s clinical condition, which supports recognition of patient deterioration (particularly sepsis) in the community, and can be used to improve the process of care and prioritise the sickest patients.^28^ The limited coding of vital signs found in this study implies that, over the study period, NEWS was not routinely being calculated in English GP practices.

Telephone consultations were also found to be associated with an increased risk of potentially missed acute deterioration. Video consultations were rarely used during the study period. Although the safety of online consulting has been questioned,^29^ there have been changes to GP appointments because of the COVID-19 pandemic, with most now conducted either by telephone or video call.^30^^,^^31^ The findings suggest that the increase to alternative consultation modes in general practice should be carefully investigated for any unintended consequences.
